# Complete Genome Sequence of a Novel Mutant of Peste des Petits Ruminants Virus Obtained from China

**DOI:** 10.1128/genomeA.01504-14

**Published:** 2015-02-12

**Authors:** Wen Su, Chao Xing, Yan Wu, Yutian Wang, Hua Ding, Hongxuan He

**Affiliations:** a National Research Center for Wildlife-Borne Diseases, Institute of Zoology, Chinese Academy of Sciences, Beijing, China; b Department of Infectious Diseases, Hangzhou Center for Disease Control and Prevention, Hangzhou, Zhejiang Province, China; c General Station of Animal Husbandry and Veterinary Medicine, Beijing, People’s Republic of China

## Abstract

Here, we announce the complete genome sequence of a spontaneous mutant of peste des petits ruminants virus (PPRV), termed China/BJ/2014, suggesting that PPRV continues to evolve. This information is crucial for developing strategies for the efficient prevention and control of PPRV.

## GENOME ANNOUNCEMENT

Peste des petits ruminants (PPR) is a viral disease of small ruminants characterized by fever, sores in the mouth, diarrhea, pneumonia, and sometimes death. PPR is an emerging global problem that adversely affects food security and poverty levels (1). The disease circulates in Africa, the Middle East, and Asia (2). In China, the situation is so bad that farmers incur heavy costs due to the prevalence of PPR (3, 4).

Peste des petits ruminants virus (PPRV) is a member of the genus *Morbillivirus* in the family of Paramyxoviridae. The genome of PPRV codes six structural proteins: nucleocapsid protein (N), phosphoprotein (P), matrix protein (M), fusion protein (F), hemagglutinin protein (H), RNA-dependent RNA polymerase (L), and two nonstructural proteins (V and C). PPRVs have been classified into four genetic lineages (I to IV) based on the comparison of complete genome sequences that are similar to the phylogenetic study using a sequence fragment of the N and/or F genes (5). Isolates from China usually belong to lineage IV and are closely related to viruses currently circulating in neighboring countries of southern Asia (3, 4).

On the second week in August 2014, most of 50 milk goats that had been vaccinated mandatorily against PPRV showed clinical signs of sore mouths, diarrhea, and weight loss. 10 of them were suspected to be dying of PPRV. Tissue samples were collected from the diseased milk goats for further characterization. Viral RNA was extracted and used directly for the presence of PPRV RNA as described previously (6, 7). The RNA from one positive lung sample was then selected for genome sequencing using a set of 13 pair primers.

Similar to a recently reported isolate, the total genome size of China/BJ/2014 is 15,954 nucleotides (nt). It is different than previously published PPRV isolates due to a 6-nt insert within the 5′ untranslated region (UTR) of the F gene (3). China/BJ/2014 has an identical genome organization as other PPRV isolates. The gene order has been established as 3′-N-P/C/V-M-F-H-L-5′ flanked by a 3′-genomic promoter and 5′-antigenomic promoter. At the nucleotide level, China/BJ/2014 shares 99.7% homology with China/XJYL/2013 (GenBank accession no. KM091959) and belongs to lineage IV according to phylogenetic analysis of complete genome sequences by MEGA 6.0. A direct comparison of China/BJ/2014 with China/XJYL/2013 demonstrated 1 mutation in P, 2 mutations in H, 4 mutations in N, 7 mutations in L, 1 mutation in C, 1 mutation in V, and no mutation in M and F. Although the role of these mutations is unclear, the data suggest that China/BJ/2014 is a mutant of China/XJYL/2013, and the virus continues to evolve and spread in China. We cannot exclude the possibility that further evolution will make preexisting vaccines increasingly ineffective against PPRV. This information is crucial for further research aimed at developing strategies for the efficient prevention and control of PPR.

### Nucleotide sequence accession number.

The complete genome of the PPRV isolate has been deposited in GenBank under the accession no. KP260624.
